# Stigma towards mental illness in med students: you label me, I label you?

**DOI:** 10.1192/j.eurpsy.2022.900

**Published:** 2022-09-01

**Authors:** C. Cabacos, A.T. Pereira, M. Carneiro, F. Carvalho, A. Manão, A. Araújo, D. Pereira, A. Macedo

**Affiliations:** Faculty of Medicine of University of Coimbra, Institute Of Psychological Medicine, Coimbra, Portugal

**Keywords:** Mental Health Stigma, psychological distress, perfectionism

## Abstract

**Introduction:**

Evidence suggests that besides having stigmatizing misconceptions towards people with mental illness, medical students and doctors often resist seeking help for their own mental issues. This is a vulnerable group for stress and other mental health problems, due not only to professional burden but also high perfectionism and low self-compassion.

**Objectives:**

To analyse the relationship between mental health stigma (MHS) and other variables related to personality and emotional states in a sample of medical students.

**Methods:**

634 medicine and dentistry students (mean age = 21.6±6.9;81.4% female) answered to a survey including sociodemographic data, self-perception of psychological health/SPPH and the Portuguese validated versions of: Link’s Perceived Discrimination and Devaluation (PDD) scale to assess MHS and its two dimensions - social stigma/SocS and self-stigma/SelS; Depression Anxiety Stress Scale (DASS-21); Neff’s Self-Compassion Scale (SCS); and Big Three Perfectionism Scale (BTPS). Correlations, t-student tests and linear regressions were performed with SPSS 27.0.

**Results:**

Stigma correlated negatively to SPPH and positively to DASS, the negative poles of SCS (self-judgement, isolation and over-identification) and BTPS second-order factors (all from p<.05 to p<.01). No gender differences in MHS were observed. Participants with higher mean levels of total and SelS had significantly higher scores in all DASS dimensions and lower SPPH; participants with higher SocS also scored higher in DASS, but didn’t reveal lower SPPH. Isolation was a significant predictor of SocS (R2=2.8%;p<.05); isolation and narcissistic perfectionism were significant predictors of SelS (R2=11%;p<.01).

**Conclusions:**

Our results highlight the importance of including MHS as a main need in the curricula of future doctors.

**Disclosure:**

No significant relationships.

